# Bilateral Sleeve Fracture of the Patella in a Healthy 11-Year-Old Male: A Case Report

**DOI:** 10.7759/cureus.50347

**Published:** 2023-12-11

**Authors:** Shintaro Usami, Takuya Naraoka, Shizuka Sasaki, Kazuki Oishi, Yasuyuki Ishibashi

**Affiliations:** 1 Orthopedic Surgery, Kuroishi General Hospital, Kuroishi, JPN; 2 Orthopedic Surgery, Hirosaki University Graduate School of Medicine, Hirosaki, JPN

**Keywords:** open reduction and internal fixation (orif), healthy child, bilateral, patella, sleeve fracture

## Abstract

Bilateral sleeve fracture of the patella (SFP) in skeletally immature children is a rare injury. We report the case of a healthy 11-year-old male who suffered bilateral SFP while playing tag. The avulsed fragments of his left patella were highly comminuted. Open reduction and internal fixation (ORIF) were performed using suture anchors, and the knees were immobilized using a cylinder cast for three weeks. At the one-year follow-up assessment, both knees were found to have regained full strength with no extension lag. However, we observed malunion due to lateral shift of the avulsed fragment, cystic lesions, and clicking in the patella, and the patient experienced residual pain in the left knee. Based on this, we conclude that the sleeve fracture of the patella with comminuted cartilaginous fragments was difficult to treat and might have led to poor clinical results if anatomical reduction and fixation had not been performed.

## Introduction

A sleeve fracture of the patella (SFP) is an injury where small osseous fragments are avulsed by the patellar tendon. In skeletally immature children before epiphyseal closure, the joints are more cartilaginous than in skeletally mature adults. SFP is a rare injury [1], and it accounts for only 0.05-0.32% of all patellar fractures in children [2,3]. Other fractures due to extensor mechanisms in skeletally immature children, such as apophyseal pelvic fractures, account for 1.4% of all fractures in young athletes [4]. SFPs represent less than 1% of all pediatric patellar fractures, and bilateral cases are even fewer. To the best of our knowledge, only two cases of bilateral SFPs in children have been previously reported in the literature [5,6].

Bilateral SFP occurs more frequently in children aged 8-11 years [3]. SFP is caused by a rapid contraction of the quadriceps as an indirect force on a flexed knee, accelerated by activities such as jumping and skateboarding [7]. SFP is diagnosed by physical tests (e.g., pain on the inferior pole of the patella) and imaging exams (e.g., plain radiographs, CT images, or MRI of the knee). SFP is usually managed with conservative treatment or open reduction and internal fixation (ORIF). Conservative treatment with a cylinder cast is performed for a minimally displaced SFP, and ORIF is performed for severely displaced SFP [8]. We present the case of a healthy 11-year-old male who suffered bilateral SFP; we also engage in a discussion about the mechanism of injury and treatment options.

## Case presentation

The patient was a healthy 11-year-old male who felt his left knee crack when he performed a take-off jump with his left leg while playing tag. He landed with his right leg, felt his right knee crack, and fell to the ground. He presented to our hospital with bilateral knee pain and difficulty walking. He had no previous medical history and no previous injury.

**Figure 1:**
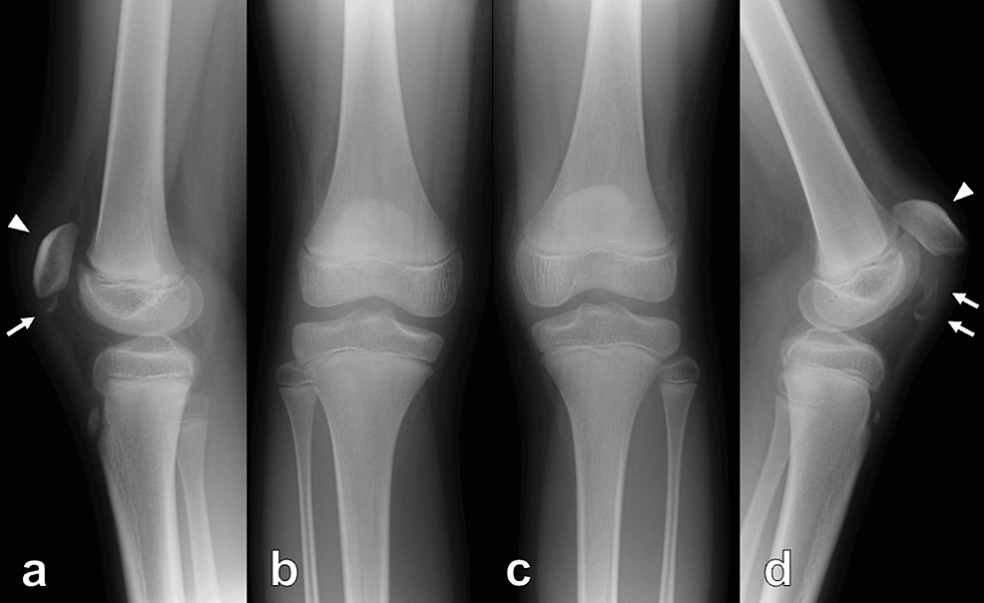
Preoperative plain radiograph of both knees Preoperative plain radiograph showing patella alta (arrowhead) and avulsed fragments of inferior poles (arrow) of both patellae (a, b: right knee; c, d: left knee)

**Figure 2:**
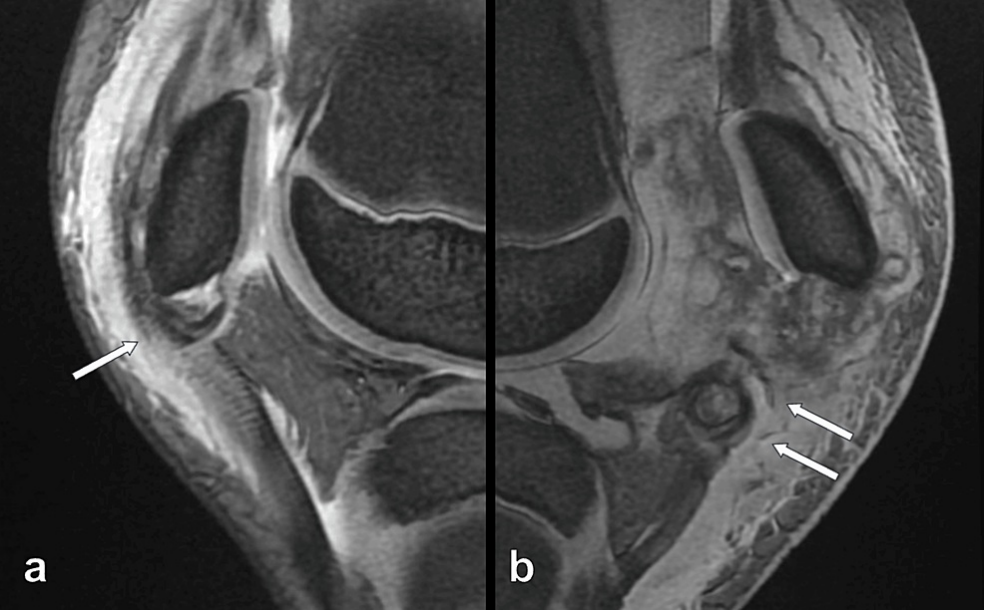
Preoperative sagittal T2-weighted MRI of both knees The image shows the avulsed fragments of inferior poles of both patellae (arrow) without patella tendon rupture (a: right knee; b: left knee) MRI: magnetic resonance imaging

His height, weight, and BMI were 152 cm, 45 kg, and 19.5 kg/m^2^, respectively. He felt pain and depression at the inferior poles of both patellae. Patellar ballottement was positive in both knees and his left knee was more swollen and more painful than his right knee. He could not do the active straight leg raising, and the extension lag test was positive. His range of motion (ROM) was +5° for extension and 30° for flexion in the right knee and +10° for extension and 30° for flexion in the left knee. A plain radiograph revealed patella alta and avulsed fragments of the inferior poles of both patellae (Figure 1). MRI showed that the distal osteochondral fragment was connected to the patellar tendon (Figure 2).

**Figure 3:**
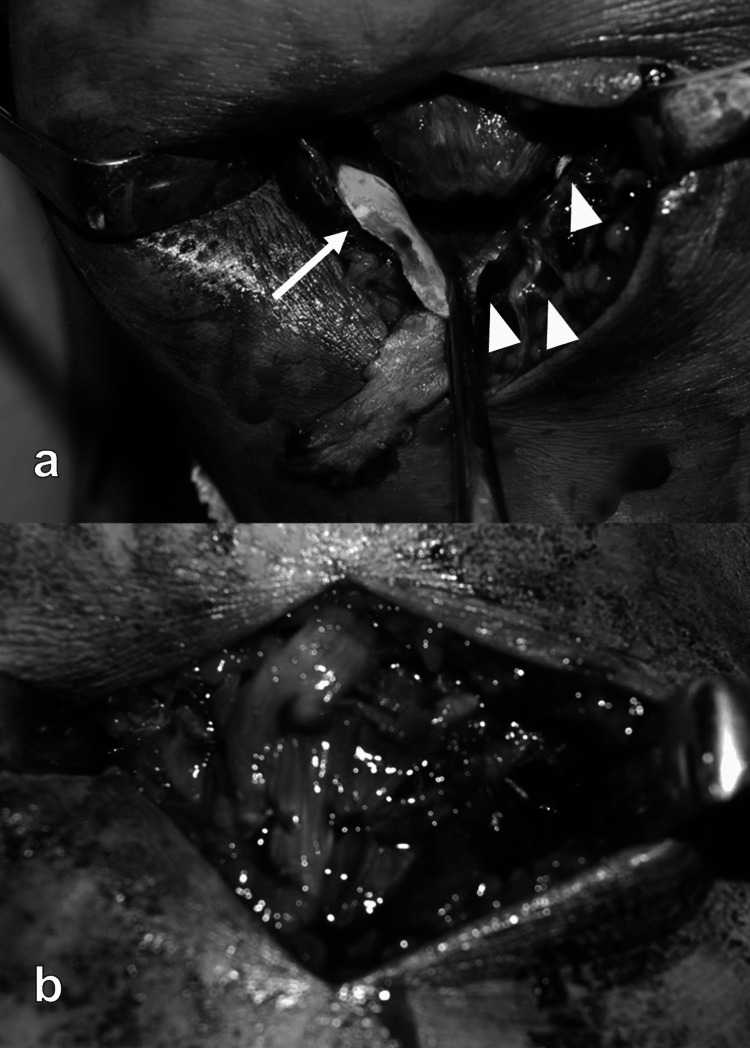
Intraoperative photographs a. Intraoperative photograph of the medial osteochondral fragment (arrow) and the lateral osteochondral fragment (arrowhead). b. The avulsed patellar tendon is sewn to the fibers of the rectus femoris overlying the patella using the Kirchmayer method

The patient underwent ORIF two days later. In his left knee, the osteochondral fragments of the inferior patellar pole attached to the patellar tendon were highly comminuted and were almost all cartilaginous (Figure 3a). After 4 anchors (Suturefix Ultra Anchor 1.9, Smith & Nephew, Andover, MA) were inserted in the fracture surface of the proximal bony fragment, the osteochondral fragments were sewn using 8 sutures. The avulsed patellar tendon was sewn to the fibers of the rectus femoris overlying the patella with #1 FiberWire® (Arthrex, Naples, FL) using the Kirchmayer method, and the medial and lateral patellar retinacula were fixed in the same way (Figure 3b). In the right knee, the fracture was also reduced and fixed with a suture anchor (Suturefix Ultra Anchor 1.9, Smith & Nephew).

**Figure 4:**
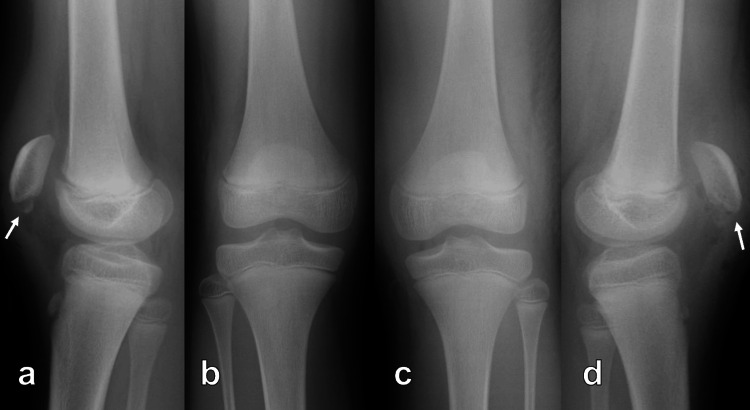
Postoperative plain radiograph of both knees Postoperative plain radiograph showing the gap between proximal bony fragment and remaining avulsed fragments (a, b: right knee; c, d: left knee)

**Figure 5:**
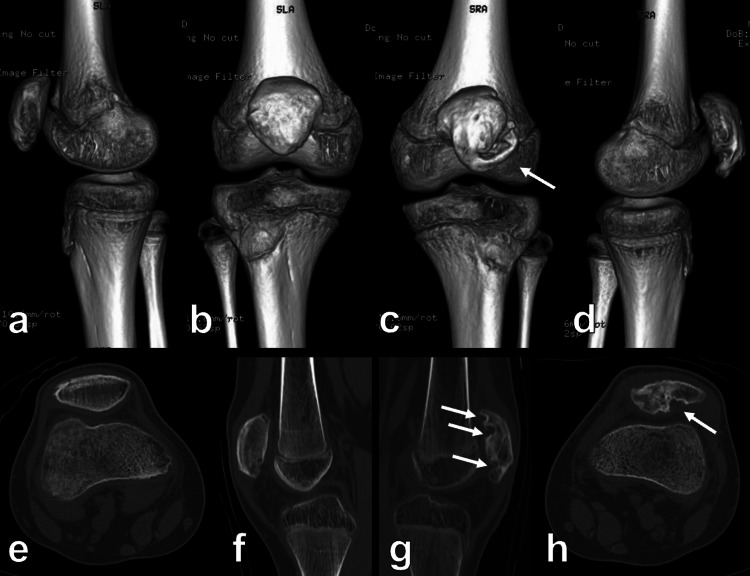
Postoperative CT of both knees at the eight-month follow-up Fragment union and good alignment are seen in the right patella (a, b, e, and f). Lateral shift of the avulsed fragment (c, d) and cystic lesions (g, h) are observed in the left patella CT: computed tomography

**Figure 6:**
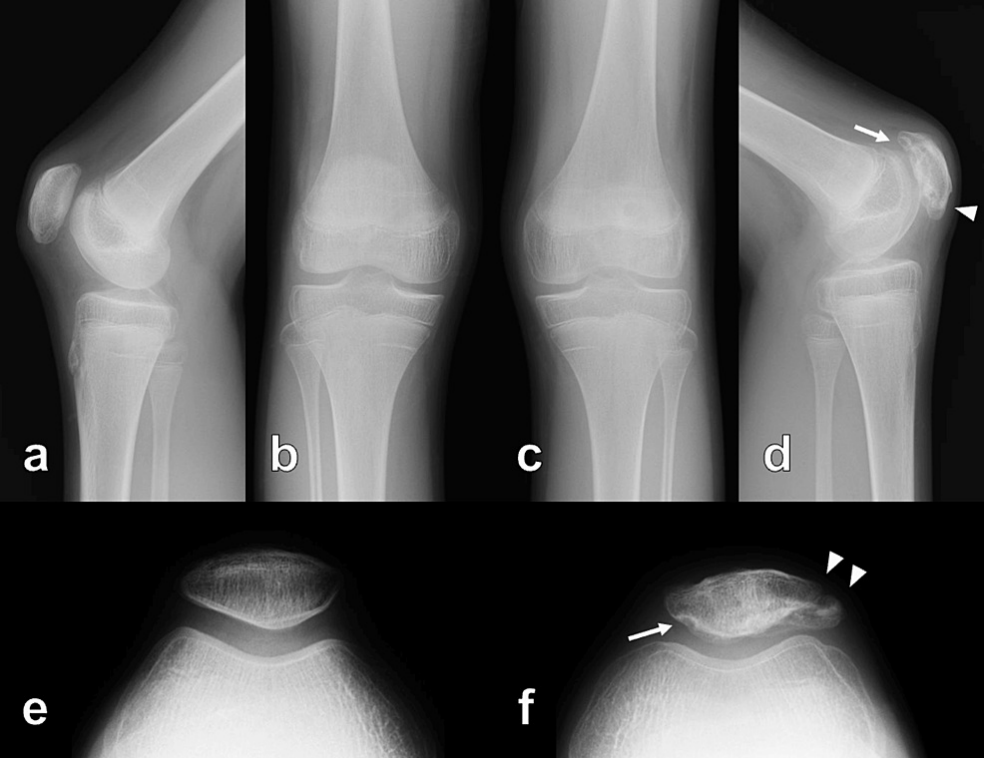
Plain radiograph of both knees at the one-year follow-up Plain radiograph at the one-year follow-up showed healed patellae without patella alta, but malunion (arrowhead) and cystic lesions (arrow) were seen in the left patella (a, b, e: right knee; c, d, f: left knee)

Postoperative plain radiographs showed that a gap remained between the proximal bony fragment and the avulsed fragment (Figure 4). Each knee was immobilized in an extended state in a cylinder cast for three weeks. ROM exercise began on week four, and full weight bearing was started on week five. At the four-month follow-up assessment, ROM in both knees was 0° for extension and 140° for flexion, and there was no extension lag in the knees. By the eight-month follow-up assessment, both knees had regained full strength with no extension lag; however, 3D CT showed a lateral shift of the avulsed fragment (Figure 5c) and cystic lesions in the left patella (Figures 5g-5h). Plain radiographs of both knees during the one-year follow-up assessment (Figure 6) showed no patella alta, and the Insall-Salvati ratio was 1.19 for the right knee and 1.06 for the left knee. The congruence angle was -5 degrees for both knees, and the tilting angle of the knee was 9 degrees for the right knee and 5 degrees for the left knee. However, the lateral shift of the avulsed fragment, cystic lesions, and the joint space narrowing of the patellofemoral joint especially in the lateral compartment were seen in the left knee. Finally, patellar malunion, clicking, and residual pain at 30° flexion were observed in the left knee.

## Discussion

This report discusses the case of a healthy 11-year-old male who suffered bilateral SFP while playing a game of tag. To the best of our knowledge, only two cases of bilateral SFP in children have been previously reported in the literature. In one of these reports, Malone et al. described bilateral SFP in a 12-year-old male with hereditary spastic paraparesis [5], and in the other report, Guy et al. discussed bilateral SFP in a healthy 11-year-old male [6]. In our case, the disorder of knee extensor mechanism or gait abnormality was not observed. Owing to the condition's rarity, the mechanism behind SFP is not completely clear and there is no established treatment for it.

SFP is an acute injury primarily resulting from forceful contraction of the quadriceps usually during jumping [1]. In earlier reports of SFP in children, the injury occurred during jumping (take-off) while playing basketball [2], high jump [9], hurdle jump [1,8], or skateboarding [1,10], landing from a jump during a basketball game [2], or in other situations such as slipping on a wet surface or from flexion-valgus stress [2]. The mechanism behind bilateral SFP was not clearly described in previous reports. Guy et al. showed that bilateral eccentric contraction of the quadriceps occurs during jumping on a trampoline [6]. In our patient, forceful concentric contraction of the left quadriceps seemed to have occurred during take-off, while forceful eccentric contraction of the right quadriceps appeared to have occurred during landing. The inferior pole of the patella is stressed maximally by the tension of the quadriceps at the mild knee flexion position, and the patella is surrounded by growth cartilage around 10 years of age [7,11]. Therefore, when patellar fractures occur in children, the patella is damaged at the dynamically weak osteochondral border [5,9].

Conservative treatment is administered using a cylinder cast for minimally displaced SFP and by ORIF for severely displaced SFP [8]. While other conservative treatment options for severely displaced SFP may lead to poor outcomes [7], ORIF is recommended because it yields good functional results [1,3,6,8,9-12]. The ORIF procedures entail the use of suture anchors [12,13], cerclage wires [6,8,10-12], tension band wiring [1,9,11], and pull-out sutures [1,6,10,11,13]. In treating SFP, the periosteum must be carefully fixed and held with lightly fastened sutures [7]. Williams et al. performed ORIF for bilateral proximal tibial sleeve fractures using Ethibond sutures, suture anchors, and cerclage wires and achieved good functional results [12]. In our patient, the avulsed fragments were comminuted and were mainly composed of cartilage; therefore, we used soft anchors during ORIF. In addition, each limb was immobilized in a cylinder cast to support the weak internal fixation. Intraoperatively, it was required to sew not only the avulsed osteochondral fragments but also the patellar tendon, but it was technically difficult to crimp the avulsed osteochondral fragments steadily to the patella. Hence, postoperative plain radiographs showed gaps between proximal bony fragments and avulsed fragments, and distal osteochondral fragments of the left patella appeared to have moved laterally. Furthermore, patellar malunion may have caused the cystic lesions due to maltracking of the patella, which produced an abnormal rotational force to the patellofemoral joint. Long-term follow-up assessment is necessary because these patients may potentially develop osteoarthritis of the patellofemoral joint in the not-distant future.

## Conclusions

We described the case of a healthy 11-year-old male who suffered bilateral SFP. In our patient, the left side SFP occurred during take-off, while the right side SFP occurred during landing. The mechanism of bilateral SFP seems to be different for each side of the knees. Because of the comminuted cartilaginous fragments, it was difficult to fix the left patella in the original position in this patient. The outcome of the conservative treatment was not reported to be less favorable, and it might have led to poor clinical results if anatomical reduction and fixation had not been performed.
